# Differential expression of miRNAs from circulating exosomes in Alzheimer disease and frontotemporal dementia

**DOI:** 10.1002/alz.091546

**Published:** 2025-01-09

**Authors:** Paulina Orellana, Ariel Caviedes, Hernan Hernandez, Patricia Lillo, Roque Villagra, Mauricio Cerda, Pedro Zitko, Daniela Thumala, Christian Gonzalez‐Billault, Agustin Ibañez, Rolando de la Cruz, Andrea Slachevsky Chonchol, Claudia Duran‐Aniotz

**Affiliations:** ^1^ Latin American Institute for Brain Health (BrainLat), Universidad Adolfo Ibañez, Santiago Chile; ^2^ Center for Social and Cognitive Neuroscience (CSCN), School of Psychology, Universidad Adolfo Ibañez, Santiago Chile; ^3^ Latin American Brain Health Institute (BrainLat), Universidad Adolfo Ibañez, Santiago Chile; ^4^ Geroscience Center for Brain Health and Metabolism (GERO), Santiago Chile; ^5^ CIMT Center for Medical Informatics and Telemedicine, School of Medicine, Universidad de Chile, santiago Chile; ^6^ Global Brain Health Institute (GBHI), Trinity College Dublin (TCD), Dublin Ireland; ^7^ Latin American Brain Health (BrainLat) Institute, Universidad Adolfo Ibáñez, Peñalolén, Santiago Chile; ^8^ Faculty of Engineering and Sciences, Universidad Adolfo Ibáñez, santiago Chile; ^9^ Neuropsychology and Clinical Neuroscience Laboratory (LANNEC), Physiopathology Department ‐ ICBM, Neuroscience and East Neuroscience Departments, Faculty of Medicine, Universidad de Chile, Santiago Chile; ^10^ Memory and Neuropsychiatric Center (CMYN), Neurology Department, Hospital del Salvador and Faculty of Medicine, Universidad de Chile, Santiago Chile

## Abstract

**Background:**

The content of circulating exosomes has been observed to be altered in response to changes in physiological and pathological conditions, and they are detectable in different human fluids such as blood. Studies focused on the quantification of Aβ and tau proteins, as molecules contained within exosomes, suggest that they are related with Alzheimer disease (AD) and frontotemporal dementia (FTD) development, demonstrated that plasma‐derived exosome analysis is a good approach for searching for biomarkers in the development of dementia. Our aim is to identify new blood biomarkers to detect the AD or FTD in the Chilean population using machine learning based on exosomal miRNAs.

**Method:**

miRNAs were extracted from circulating exosomes from plasma samples of 25 healthy controls (HC), 25 AD patients and 10 FTD patients. miRNAs were sequencing to identify and measure the expression levels. Dysregulated miRNAs were analyzed to create machine learning algorithms. miRNAs obtained from algorithms were validated in a new population of subjects 30 HC, 30 AD and 10 FTD using qRT‐PCR.

**Result:**

After the sequencing and machine learning analysis, we identified 8 miRNAs as potential predictors of AD and FTD. These miRNAs were further assessed in new samples using the qRT‐PCR technique. We identified 4 miRNAs with variable expression in patients with AD and FTD, with three miRNAs downregulated in AD and one upregulated in FTD. For the four miRNAs, significant differences in expression are observed between AD and FTD.

**Conclusion:**

This is the first study in the Chilean population that evaluates miRNAs in patients with AD and FTD. The study of miRNAs as biomarkers of dementia provides the opportunity to develop low‐cost and easy‐to‐obtain diagnostic methods, in addition to the possibility of being implemented in a greater number of health centers, making them more accessible to the population.

